# Interleukin 21 Receptor/Ligand Interaction Is Linked to Disease Progression in Pancreatic Cancer

**DOI:** 10.3390/cells8091104

**Published:** 2019-09-18

**Authors:** Alica Linnebacher, Philipp Mayer, Nicole Marnet, Frank Bergmann, Esther Herpel, Steffie Revia, Libo Yin, Li Liu, Thilo Hackert, Thomas Giese, Ingrid Herr, Matthias M. Gaida

**Affiliations:** 1 Institute of Pathology, University Hospital Heidelberg, 69120 Heidelberg, Germany; alica.linnebacher@med.uni-heidelberg.de (A.L.); nicole.marnet@med.uni-heidelberg.de (N.M.); pathologie@mail.klinikum-darmstadt.de (F.B.); Esther.Herpel@med.uni-heidelberg.de (E.H.);; 2 Clinic for Diagnostic and Interventional Radiology, University Hospital Heidelberg, 69120 Heidelberg, Germany; Philipp.Mayer@med.uni-heidelberg.de; 3 Clinical Pathology, Klinikum Darmstadt GmbH, 64283 Darmstadt, Germany; 4 Helmholtz-University Group “Cell Plasticity and Epigenetic Remodeling”, German Cancer Research Center (DKFZ) and Institute of Pathology, University Hospital, 69120 Heidelberg, Germany; s.revia@dkfz-heidelberg.de; 5 Molecular OncoSurgery Group, Department of General, Visceral, and Transplantation Surgery, University of Heidelberg and German Cancer Research Center, 69120 Heidelberg, Germany; yin@uni-heidelberg.de (L.Y.); l.liu@uni-heidelberg.de (L.L.); i.herr@uni-heidelberg.de (I.H.); 6 Department of General, Visceral, and Transplantation Surgery, University Hospital Heidelberg, 69120 Heidelberg, Germany; thilo.hackert@med.uni-heidelberg.de; 7 Institute of Immunology, University Hospital Heidelberg, 69120 Heidelberg, Germany; Giese@uni-hd.de; 8 Institute of Pathology, University Medical Center Mainz, 55131 Mainz, Germany

**Keywords:** pancreatic cancer, tumor microenvironment, IL-21, Blimp-1, Th17, invasion

## Abstract

Pancreatic ductal adenocarcinoma (PDAC) displays a marked fibro-inflammatory microenvironment in which infiltrated immune cells fail to eliminate the tumor cells and often—rather paradoxically—promote tumor progression. Of special interest are tumor-promoting T cells that assume a Th17-like phenotype because their presence in PDAC tissue is associated with a poor prognosis. In that context, the role of IL-21, a major cytokine released by Th17-like cells, was assessed. In all tissue samples (n = 264) IL-21^+^ immune cells were detected by immunohistochemistry and high density of those cells was associated with poor prognosis. In the majority of patients (221/264), tumor cells expressed the receptor for IL-21 (IL-21R) and also a downstream target of IL-21, Blimp-1 (199/264). Blimp-1 expression closely correlated with IL-21R expression and multivariate analysis revealed that expression of both IL-21R and Blimp-1 was associated with shorter survival time of the patients. In vitro data using pancreatic tumor cells lines provided a possible explanation: IL-21 activated ERK and STAT3 pathways and upregulated Blimp-1. Moreover, IL-21 increased invasion of tumor cell lines in a Blimp-1-dependent manner. As an in vivo correlate, an avian xenograft model was used. Here again Blimp-1 expression was significantly upregulated in IL-21 stimulated tumor cells. In summary, our data showed an association of IL-21^+^ immune cell infiltration and IL-21 receptor expression in PDAC with poor survival, most likely due to an IL-21-mediated promotion of tumor cell invasion and enhanced colony formation, supporting the notion of the tumor-promoting abilities of the tumor microenvironment.

## 1. Introduction

Pancreatic ductal carcinoma (PDAC) is a highly aggressive tumor with a 5-year survival time of about 9% [1]. Characteristic for PDAC is the marked fibro-inflammatory microenvironment consisting of a fibrotic desmoplastic stroma that represents a major part of the tumor mass. Interspersed are pancreatic stellate cells and myofibroblasts and infiltrated immune cells, including neutrophils, macrophages, and T cells. Apparently, the immune cells fail to eliminate the tumor, but —paradoxically—may contribute to tumor progression [2,3]. Dense neutrophil infiltrates, for example, are associated with aggressive disease, possibly due to neutrophil-derived enzymes that induce epithelial–mesenchymal transition, migration and invasion of tumor cells, or modulation of the stroma [4,5,6,7].

Presence of T cells in PDAC has been reported in numerous publications, with conflicting results. Ino et al. reported a better prognosis of PDAC patients with high density of total T cells [8], whereas others state that high numbers of Th17 cells are associated with rapid disease progression [9]. A possible explanation is that not only the number of T cells but also the subclass and their cytokine profile is of significance. In a previous work, one of us (MMG) found tumor infiltrating T cells in human PDAC, particularly CD4 (helper) T cells, producing tumor necrosis factor (TNF)-α and interleukin (IL)-17A. High density of those T cells was associated with poor survival [10]. Interestingly, these patients also have high numbers of IL-21 positive T cells, although the function of IL-21 and its receptor in human pancreatic cancer was unclear.

IL-21 is a pleiotropic cytokine that is mainly produced by activated T cells, particularly by the pro-inflammatory Th17 subset. It modulates T cell activation [11,12] and it is crucially involved in B cell maturation [13,14] and natural killer (NK) cell expansion [15]. IL-21 activates its target cell via a receptor, a heterodimer consisting of the IL-21 receptor (IL-21R) and the so-called common gamma chain (CD132), the latter expressed by nearly all immune cells. As downstream signaling pathways, the JAK/STAT pathway, the phosphoinositide 3-kinase/protein kinase B (PI3K/AKT), and the ras/raf/Mitogen-activated protein kinase kinase (MEK)/mitogen-activated protein kinases (MAPK) pathways have been identified [16,17,18,19]. A relevant downstream target of IL-21 is the B lymphocyte-induced maturation protein-1 (Blimp-1) that in humans is encoded by the *PRDM1* gene [20,21,22]. Other known downstream targets include GATA3 [23] or Bcl-6 [24].

The role of IL-21 in tumor biology is controversially discussed. Mainly anti-neoplastic effects attributed to enhanced expansion, cytotoxicity, and activation of CD8^+^ T cells and NK cells were described [25,26]. In particular, an increased production of granzymes, cytotoxic molecules of T cells and NK cells, was shown, as was enhanced IFN-γ production, the latter a potent activator for NK cells [16,18,27]. Moreover, transduction of IL-21 constructs into pancreatic cancer cell lines resulted in anti-tumor effects when the cells were implanted into T cell-free NOD/SCID mice [28]. A clinical study for non-progressed melanoma showed a partial response or disease stabilization in 20% of patients [29], but definite results are pending.

A few studies, in contrast, linked IL-21 with inflammatory colon carcinogenesis, tumor development or tumor progression [30,31,32,33]. Furthermore, in breast cancer, IL-21 enhanced tumor cell proliferation and induced matrix metalloproteinases, the latter known to participate in tumor invasion [34].

The discrepant findings could be due to different tumor entities or due to different experimental approaches. Especially the studies with tumors implanted into immune-incompetent animals may underestimate the role of the inflammatory environment present typically in PDAC.

Hence, to evaluate the role of IL-21 in human pancreatic cancer, in the present study we analyzed tissue specimen of patients with PDAC and in vitro experiments with pancreatic cell lines as well as an avian xenograft model as an in vivo correlate. 

In this study, IL-21^+^ immune cell infiltration and IL-21 receptor expression in PDAC could be associated with poor survival. Furthermore, an IL-21-mediated promotion of tumor cell invasion could be shown in vitro, supporting the notion of the tumor-promoting abilities of cytokines, released by inflammatory cells of the tumor microenvironment.

## 2. Materials and Methods

### 2.1. Patient Samples and Immunohistochemistry

Tissue samples were obtained from the tissue bank of the National Center for Tumor Diseases (NCT, Heidelberg, Germany) in accordance with the regulations of the tissue bank and the approval of the ethics committee of Heidelberg University (no. 206/2005). A written informed consent of all patients was obtained.

Tissue samples of 264 patients with pancreatic ductal adenocarcinoma who underwent surgical resection with curative intent were analyzed as microarrays. Paraffin-embedded tissue was used. For immunohistochemical analysis using the following antibodies: rabbit anti-human Blimp-1 (1:50; Cell Signaling Technology, Leiden, Netherlands), rabbit anti-human IL-21 receptor (1:50; Novus Biologicals, Bio-Techne GmbH, Wiesbaden, Germany), rabbit anti-human IL-21 (1:100; Abcam, Cambridge, UK), mouse anti-human GATA3 (ready to use; Roche, Mannheim, Germany), rabbit anti-human RORC (1:100, LifeSpan BioSciences, Eching, Germany). Antigen retrieval was performed by heat pre-treatment using citrate buffer (pH 6.0) and antibody-binding was visualized by the avidin-biotin complex method (EnVision, Dako, Glostrup, Denmark) or with liquid permanent red (Zytomed, Berlin, Germany). The presence of the respective antigens was semi-quantified using the well-established Allred score [35].

### 2.2. Cloning

All primers and guide sequences used for cloning are listed in Supplementary Tables S1 and S2.

CRISPR/Cas9: pLenti-Blimp-1-Puro was generated by annealing and phosphorylation of the single stranded guide RNA against *PRDM1* which is then ligated into a BsmBI-digested pLenti-CRISPR v2 backbone.

Overexpression: pTRIPZ-Blimp-1-Puro was generated by Gibson assembly, combining a PCR-amplified *PRDM1* cDNA from RGS-6xHis-BLIMP-1-pcDNA3.1 (52518, addgene), with an AgeI/MluI-digested pTRIPZ backbone.

### 2.3. Cell Culture, Transfection, and Transduction

Cell culture: The human PDAC cell lines AsPC-1, BxPC-3, and Panc-1 were obtained from ATCC and cultivated in RPMI 1640 (Life Technologies GmbH, Darmstadt, Germany) supplemented with 10% FBS and 1% penicillin and streptomycin (P/S). HEK293T (ATCC CRL-3216) cells were maintained in Dulbecco’s modified Eagle’s medium (Life Technologies GmbH) supplemented with 10% FBS and 1% P/S. All cells were incubated at 37 °C, with 5% CO_2_ and 95% humidity. For the experiments, cells were harvested when in linear growth condition. Details are described in the respective experiment.

Transient transfection (for *PRDM1* siRNA knockdown): Pancreatic cancer cells BxPC-3 and Panc-1 in a 6-well plate were transfected with 10 nM of a universal scrambled control siRNA (SR30004; OriGene) or three individual *PRDM1*-targeting siRNAs (SR300437A, SR300437B, SR300437C; OriGene) in Opti-MEM using siTran 1.0 siRNA transfection reagent (TT300001; OriGene) according to the manufacturer’s instructions. Cells were maintained in Opti-MEM for 4–6 h post transfection and then incubated in RPMI with 10% FBS and 1% P/S. Based on knockdown of Blimp-1 on protein level after transfection with the different siRNAs, SR300437C was chosen as the most efficient siRNA (reduction: BxPC-3: 69%, Panc-1: 51%) for further experiments.

Transfection (for virus production): HEK293T cells were plated in 10-cm dishes and transfected 24 h later (95% confluence) with a prepared mix in DMEM media (no supplements) containing 10 μg of lentiviral backbone, 8 μg of PAX2, 2.5 μg of VSV-G, and 60 μL of PEI (1 mg/mL). 24 h following transfection, media was replaced with target cell collection media and supernatants were harvested 48 and 56 h post transfection.

Transduction: 0.3 × 10^6^ BxPC-3 and Panc-1 cells were plated on 6-well plates. 24 h following plating, cells were transduced with viral supernatants in the presence of polybrene (8 μg/μL). Two days after transduction cells were selected in puromycin (2 µg/mL).

### 2.4. Western Blotting

For western blotting experiments, cells were seeded at a density of 80 to 90%. Recombinant human IL-21 (200-21; Peprotech GmbH, Hamburg, Germany) was added for the times indicated, as was the ERK-inhibitor (30 µM; sc-203945; Santa Cruz Biotechnologies, Heidelberg, Germany).

Cells were harvested and lyzed with RIPA buffer (Santa Cruz Biotechnologies) and total protein content of solubilized material was quantified by Pierce BCA Protein Assay Kit (Thermo Fisher, Dreieich, Germany). Of the lysate, 20 µg was subjected to SDS-PAGE (10%) and Prestained Rec Protein Ladder (Thermo Fisher) was used as a marker.

For western blotting, proteins were transferred to a 0.45 μm nitrocellulose membrane (GE Healthcare GmbH, Freiburg, Germany) and 5% skim milk (Carl Roth GmbH, Karlsruhe, Germany) or 3% BSA (Sigma-Aldrich GmbH, Taufkirchen, Germany) were used for blocking. The following primary antibodies were used: rabbit anti-human IL-21R (NBP1-76739; Novus Biologicals), rabbit anti-human Blimp-1 (9115S; Cell Signaling Technology), rabbit anti-human ERK (9102S; Cell Signaling Technology), rabbit anti-human pERK (9101S; Cell Signaling Technology), rabbit anti-human STAT3 (4904S; Cell Signaling Technology), rabbit anti-human pSTAT3 (9145S; Cell Signaling Technology), rabbit anti-human p38 (9212S; Cell Signaling Technology), rabbit anti-human pp38 (4631S; Cell Signaling Technology), and mouse anti-human β-tubulin (sc-5274; Santa Cruz Biotechnologies). Following incubation at 4 °C overnight, antibody binding was assessed using fluorescent donkey anti-rabbit or donkey anti-mouse secondary antibodies (LI-COR® Biosciences GmbH, Bad Homburg, Germany). Protein bands were visualized using a LI-COR® Imaging Systems (LI-COR® Biosciences GmbH) and documented by LI-COR® Image Studio™ Software. For each experimental condition, at least three independent experiments were performed.

### 2.5. Quantitative RT-PCR (qRT-PCR)

Instruments and reagents for qRT-PCR were purchased from Roche Applied Biosciences AG (Mannheim, Germany) and applied as described previously [10,36]. In brief, mRNA was extracted using the MagNA Pure^®^ System for cells; cDNA was synthesized with the First Strand cDNA synthesis kit and the PCR was conducted using the LightCycler^®^480. Primers were obtained from Search-LC (Heidelberg, Germany). The Blimp-1 copy numbers were normalized to the housekeeping gene peptidylprolyl isomerase B (PPIB) and are expressed as the number of specific transcripts per 10,000 PPIB copies.

### 2.6. Immunofluorescence Staining of IL-21R

Pancreatic cancer cells (50 × 10^3^/500 µL) were seeded into Lab-Tek® Chamber Slides (Thermo Fisher). After 24 h, cells were fixed with 2% PFA and incubated with a rabbit anti-human IL-21R antibody (NBP1-76739; Novus Biologicals) at 4 °C overnight. As a secondary antibody, Alexa488-conjugated goat anti-rabbit antibody (Dianova, Jackson, Hamburg, Germany) was used. Slides were mounted with a coverslip using ProLong® Diamond Antifade Mountant with DAPI (Thermo Fisher) and viewed by digital microscopy (Keyence, Neu-Isenburg, Germany).

### 2.7. Cell Proliferation Assay

Cell proliferation was measured using a BrdU Cell Proliferation Assay kit (6813; Cell Signaling) following the manufacturer’s instructions. Absorbance was read at 450 nm using the Omega Fluostar Microplate Reader (BMG Labtech, Ortenberg, Germany).

### 2.8. In Vitro Matrigel Invasion Assay

Corning® BioCoat™ Matrigel® Invasion Chambers with 8.0 μm PET Membrane (Corning GmbH, Wiesbaden, Germany) were rehydrated with RPMI in a 24-well plate for 2 h at 37 °C and 5% CO_2_. A subset of cells, transfected with a pTRIPZ EV or pTRIPZ Blimp-1 construct for doxycycline (dox)-inducible overexpression experiments, was treated with or without dox (1 µg/mL) 48 h prior to and during the experiment. Cells (2.5 × 10^4^ in 500 µL) were seeded on top of the inserts and IL-21 added to the bottom wells in a final concentration of 10 ng/mL. After 48 h, non-invaded cells from the upper surface were removed. Invaded cells at the lower surface were fixed with 100% ice-cold methanol for 20 min at −20 °C and stained with Mayer´s Hemalaun solution (AppliChem, Darmstadt, Germany) for 15 min at RT, mounted to coverslips and counted under a microscope. For each experimental condition, at least four independent experiments were performed.

### 2.9. Mutation Detection by T7 Assay

Primers used for T7 endonuclease assay are listed in Supplementary Table S3.

Cas9-induced mutations were detected using the T7 endonuclease I (M0302L, New England Biolabs (NEB), Frankfurt, Germany), as previously described [37]. Briefly, an approximately 800 bp region surrounding the expected mutation site was PCR-amplified using the Q5^®^ High-Fidelity DNA Polymerase (M0491L, NEB). PCR products were column purified (Qiagen) and subjected to a series of melt–anneal temperature cycles with annealing temperatures gradually lowered in each successive cycle. T7 endonuclease I was then added to selectively digest heteroduplex DNA. Digest products were visualized on a 2% agarose gel.

### 2.10. Chick Chorio-Allantoic Membrane Assay: Treatment of Avian Xenografts with IL-21 and Immunohistochemistry

This assay was performed as previously described [38] with the following modifications. Fertilized white Leghorn chicken eggs (Geflügelzucht Hockenberger, Eppingen, Germany) were incubated at a humidity of 45–55% at 37.8 °C in digital motor breeders Type 168/D (Siepmann GmbH, Herdecke, Germany). At day 4 of embryonic development, 2–3 mL of albumen was removed with a syringe to detach the embryo. A small incision was made into the eggshell and sealed with tape. At day 9 of embryonic development, small handmade rings from Thermanox™ cover discs (Thermo Scientific, Schwerte, Germany) were placed on the chorioallantoic membrane (CAM) and 5 × 10^5^ tumor cells (BxPC-3) in 50 µL Matrigel were deposited into the rings. At days 12 and 16 of embryonic development, 50 µL of IL-21 (150 ng/mL) were injected intravenously. Chick saline was used as a vehicle control. At day 17, the embryos were euthanized and tumor size was evaluated.

Tumors were resected and tumor volumes were determined by the following formula: Volume = 4/3 × π × *r*^3^ (*r* = ½ × square root of diameter 1 × diameter 2) [39]. Then H&E staining and immunohistochemistry was performed. The following antibodies were used: mouse anti-human Ki67 (1:400, pH 9; Dako) to detect proliferating cells, rabbit anti-human FVIII (1:1000, pH 6; Dako) to detect neovascularization, rabbit anti-human IL-21R (1:50, pH 6; Novus Biologicals), and rabbit anti-human Blimp-1 (1:50, pH 6; Cell Signaling Technology).

Slides were scanned at 400× using an Aperio CS scanner (Leica Biosystems GmbH, Wetzlar, Germany) and stored in the Aperio svs file format, using JPEG compression in eSlide Manager (Aperio) and evaluated using ImageScope Software v12.3.2.8013 (Aperio).

### 2.11. Statistical Analysis

Statistical data analysis was performed using MedCalc Version 18.10.2 (MedCalc Software, Ostend, Belgium). Pearson correlation coefficients were calculated a) between Blimp-1 and IL-21R Allred scores, b) between IL-21 infiltrate and Blimp-1 Allred score/IL-21R Allred score, c) between Blimp-1 Allred score/IL-21R Allred score/IL-21 infiltrate and clinico-pathological parameters (T [extent of primary tumor], N [regional lymph node metastases], G [grading]).

Overall survival (OS) analyses were performed using Kaplan–Meier estimates and Cox regression. Patients who died within the first 60 days after surgery were excluded from the OS analyses since complications from surgery were considered the most probable cause of death. Follow-up data ≥60 days regarding OS were available from 213 patients with PDAC.

OS curves were estimated with the Kaplan–Meier method for patients with low Blimp-1 Allred scores (0 to 3) versus patients with high Blimp-1 Allred scores (4 to 8), for patients with low IL-21R Allred scores (0 to 3) versus patients with high IL-21R Allred scores (4 to 8), as well as for patients with low IL-21 infiltrate (<12 IL-21^+^ cells/mm^2^) versus patients with high IL-21 infiltrate (≥12 IL-21^+^ cells/mm^2^). Survival distributions of the samples were compared by two-sided log-rank test.

Additionally, the Cox proportional hazard model was used for multivariate analysis. Predictor variables were T (extent of primary tumor, T1 vs. T2 to T3), N (regional lymph node metastases, N0 vs. N+), G (grading, G1 to 2 vs. G3), Blimp-1 Allred score (score 0 to 3 vs. score 4 to 8), IL-21R Allred score (score 0 to 3 vs. score 4 to 8), and IL-21 infiltrate (<12 IL-21^+^ cells/mm^2^ vs. ≥12 IL-21^+^ cells/mm^2^). Forward selection method was applied. A variable was entered into the model if its associated significance level was <0.05. A variable was removed from the model if its associated significance level was >0.1.

*P* values < 0.05 were considered to be statistically significant.

## 3. Results

### 3.1. IL-21, IL-21 Receptor and Blimp-1 in Pancreatic Cancer Tissue and Association with Clinical Data

**Figure 1:**
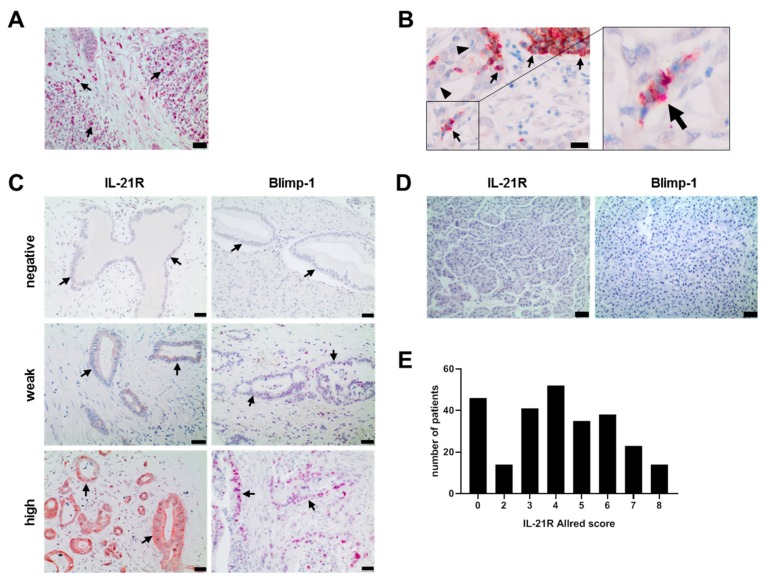

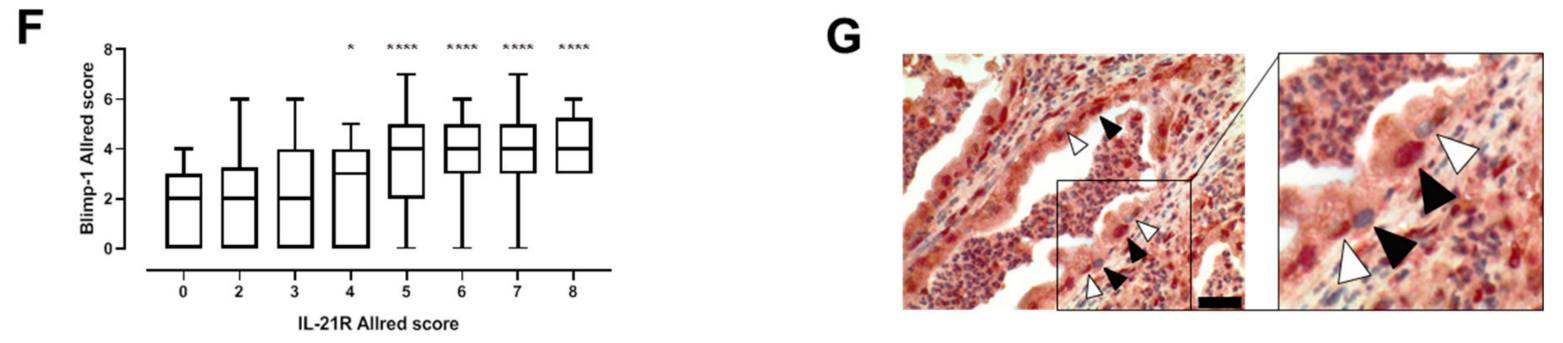
IL-21R, IL-21, and Blimp-1 expression in human PDAC and healthy pancreas tissue samples. (**A**) Infiltration of IL-21^+^ immune cells (red) in PDAC. Black arrow: IL-21^+^ cells. (**B**) Infiltration of IL-21^+^ Th17 cells in PDAC: IL-21^+^ (red) and RORC^+^ (brown) cells. Black arrowhead: PDAC tumor cells, black arrow: IL-21^+^/RORC^+^ immune cells. (**C**) Examples of negative, weak and high expression of IL-21R (brown) and Blimp-1 (red) in human PDAC tissue. Black arrows: tumor cells. (**D**) Examples of IL-21R and Blimp-1 expression in healthy pancreas tissue. (**E**) IL-21R expression in human PDAC varied among the patients. (**F**) Association of IL-21R and Blimp-1 expression in human PDAC. * *P* < 0.05 and **** *P* < 0.0001. Unpaired *t*-test. (**G**) Co-expression of IL-21R (brown, membrane) and Blimp-1 (red, nucleus). Black arrowhead: IL-21R^+^/Blimp-1^+^, white arrow head: IL-21R^+^/Blimp-1^−^. Black bar: 20 µm.

**Figure 2:**
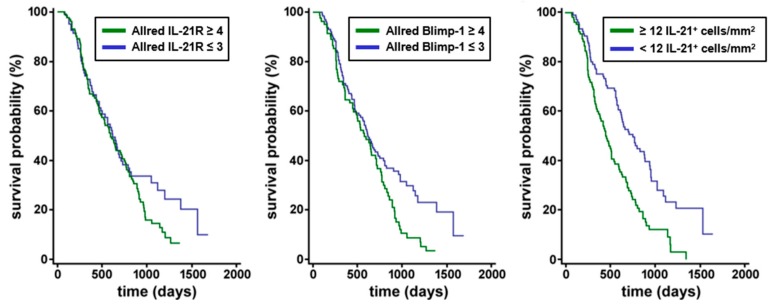
Correlation of IL-21 receptor with Blimp-1 expression and effect on overall survival in human PDAC biopsies. Survival analysis of IL-21R and Blimp-1 expression. There was a non-significant trend towards shorter OS in PDAC patients with high IL-21R expression compared to patients with low IL-21R expression. PDAC patients expressing a high level of Blimp-1 had a significantly shorter survival compared to patients expressing a low level of Blimp-1. Survival was significantly shorter in patients with high IL-21 infiltrate compared to patients with low IL-21 infiltrate.

IL-21^+^ and IL-21^+^RORC^+^ Th17 cells were found in tissue of all patients (n = 264) though to a varying degree (Figure 1A,B). Dense IL-21 infiltrates (≥12 IL-21^+^ cells/mm^2^) were associated with shorter survival time (median 462 days) when compared to patients with low IL-21 infiltrate <12 IL-21^+^ cells/mm^2^ (median 771 days, *p*-value of log-rank test <0.0001) (data summarized in Figure 2).

In the same tissue specimen, expression of IL-21R and its downstream target Blimp-1 was determined. IL-21R was found to be expressed on tumor cells of 221/264 patients and Blimp-1 in 199/264, but not in healthy pancreatic tissue (Figure 1C,D). Expression of IL-21R varied among the patients, as quantified by Allred score (distribution shown in Figure 1E) and high Blimp-1 expression coincided significantly with high IL-21R expression (Figure 1F, unpaired t test and Pearson correlation test (r = 0.5604, *p* < 0.0001)). Double staining of the tissue revealed a co-expression of IL-21R and Blimp-1 (Figure 1G).

A weak but significant positive Pearson’s correlation between the Blimp-1 Allred score and the IL-21 infiltrate (r = 0.1828, *p* = 0.0079) was seen, but no correlation between the IL-21R Allred score and the IL-21 infiltrate. IL-21 infiltrate did not correlate with clinico-pathological parameters (T, N, G).

There was a trend towards shorter overall survival (OS) time in patients with high IL-21R Allred scores (score 4 to 8, median 592 days) compared to patients with low IL-21R Allred scores (scores 0 to 3, median 616 days), although the difference was not statistically significant according to the log-rank test (*p* = 0.1516). The OS time of patients with high Blimp-1 expression (Allred scores score 4 to 8) was considerably shorter compared to patients with low Blimp-1 Allred scores (scores 0 to 3) as assessed by Kaplan–Meier-Analysis (576 days versus 616 days; log-rank test *p* = 0.0126) (data summarized in Figure 2).

Applying the Cox regression model, the following factors were significantly associated with impaired OS: high Blimp-1 Allred scores (scores 4 to 8) compared low Blimp-1 Allred scores (scores 0 to 3) (hazard ratio (HR), 1.3804; 95% confidence interval (CI), 1.0066 to 1.8931; *p* = 0.0454), high IL-21 infiltrate (≥12 IL-21^+^ cells/mm2) compared to low IL-21 infiltrate (<12 IL-21^+^ cells/mm2) (HR, 2.0213; 95% CI 1.4650 to 2.7888, *p* < 0.0001), intermediate or high T-stage (T2 or T3) compared to low T-stage (T1) (HR, 1.9083; 95% CI, 1.1455 to 3.1793, *p* = 0.0131) and high grading (G3) compared to low or intermediate grading (G1 or G2) (HR, 1.6146; 95% CI, 1.1693 to 2.2295; *p* = 0.0036). Lymph node positivity (N+, compared to N0) and high IL-21R Allred scores (scores 4 to 8, compared to scores 0 to 3) were found to not significantly contribute to OS.

GATA3, a further transcription factor of interest, was also expressed in PDAC tissue, but not on healthy pancreatic tissue (Supplementary Figure 1A,B). GATA3 expression correlated with Blimp-1 expression (quantified by Allred score; r = 0.2423; *p* = 0.0012) and with expression of IL-21R (r = 0.1515; *p* = 0.0453). There was no correlation between GATA3 expression and IL-21^+^ cells. Patients with GATA3 Allred score ≥4 had a shorter survival compared with GATA3 Allred score <4 (592 d versus 616 d; *p* = 0.2762, log rank test), though this was a rather minor effect (data not shown).

### 3.2. IL-21R on Pancreatic Tumor Cell Lines and Upregulation of Blimp-1

**Figure 3:**
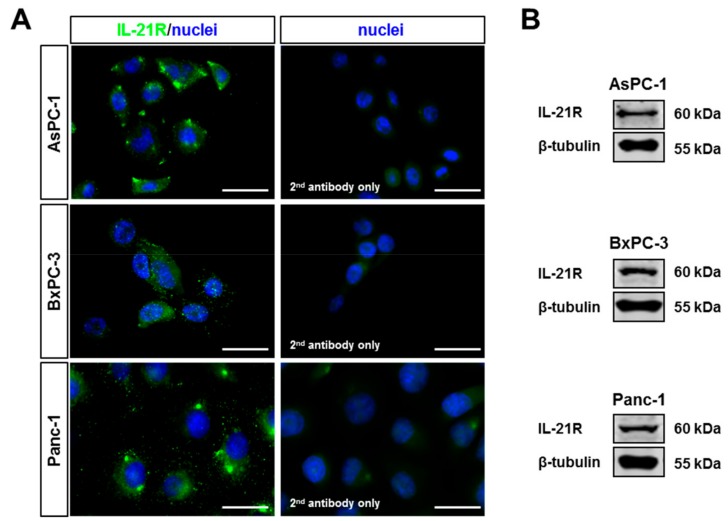
IL-21R expression in human PDAC cell lines. Untreated human PDAC cell lines AsPC-1, BxPC-3, and Panc-1 were tested for IL-21R expression in (**A**) immunofluorescence staining (left: IL-21R^+^ cells, right: Alexa488 goat anti-rabbit IgG only; nuclei: DAPI (blue), IL-21R: Alexa488 (green)) and (**B**) western blot. White bar: 20 µm.

**Figure 4:**
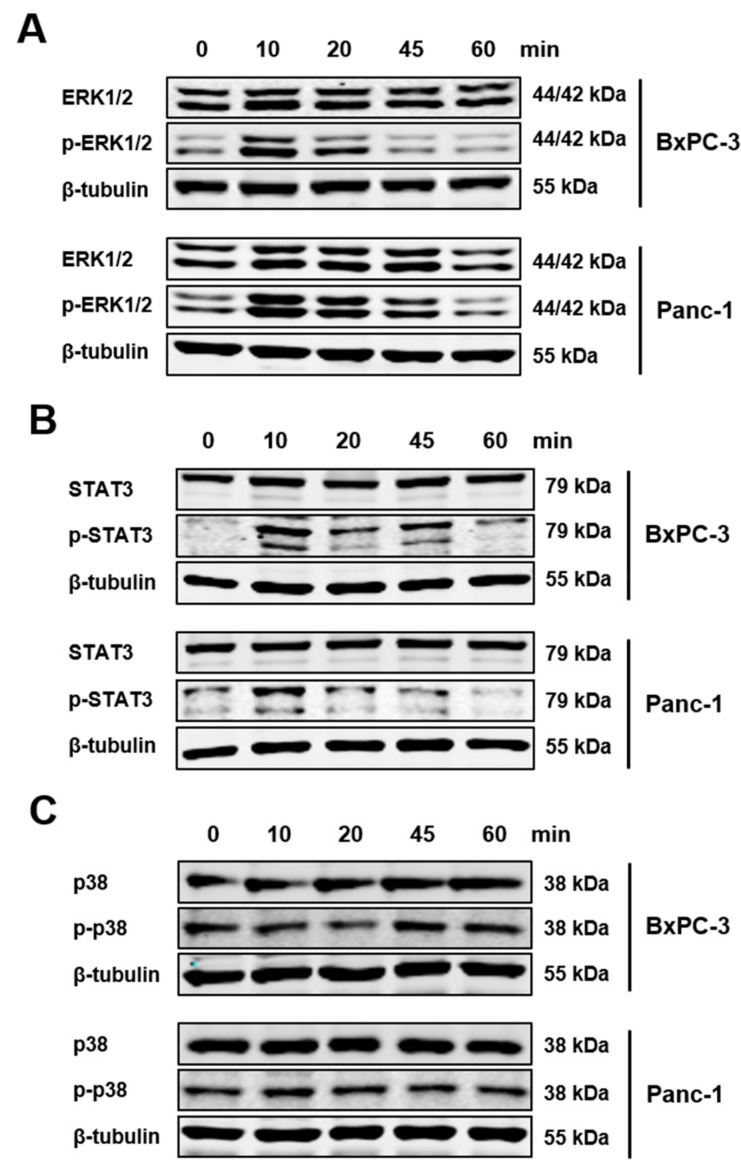
IL-21R activates ERK and STAT3 pathway; but not p38. PDAC cells BxPC-3 and Panc-1 were incubated with IL-21 (10 ng/mL) for the time indicated. Phosphorylation of (**A**) ERK, (**B**) STAT3, and (**C**) p38 was evaluated. β-tubulin was used as a loading control. After 10–20 minutes, phosphorylation of ERK and STAT3 could be detected. One of three independent experiments is shown.

The tissue analysis implied that tumor cells acquire the receptor for IL-21. Therefore, three pancreatic tumor cell lines (AsPC-1, BxPC-3, and Panc-1) were tested for expression of IL-21R by western blotting and immunofluorescence staining. All expressed IL-21R (Figure 3A,B). For further analysis, Panc-1, and BxPC-3 were chosen, as examples for cells derived from a non-metastatic and a metastatic tumor, respectively. Triggering the receptor with IL-21 resulted in phosphorylation of ERK and STAT3, whereas p38 was not affected (Figure 4).

**Figure 5:**
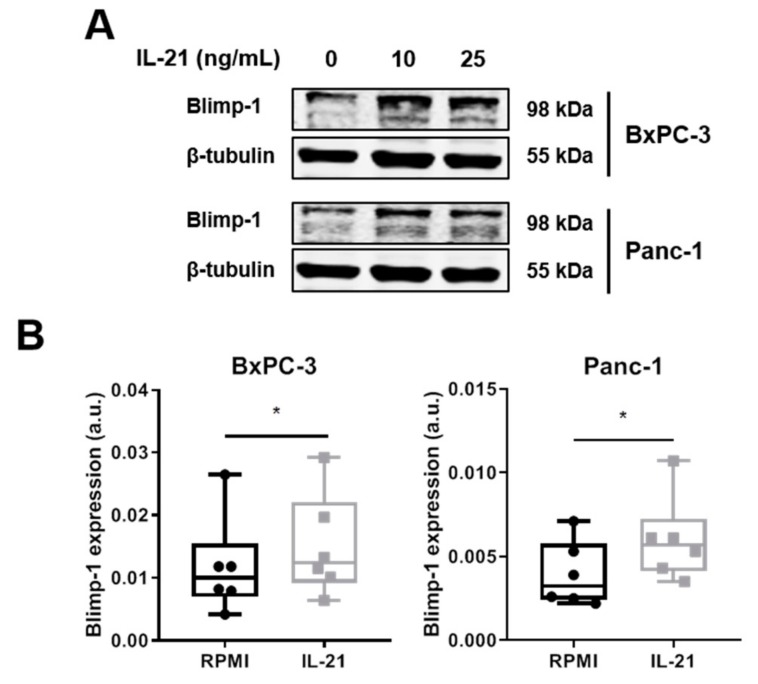

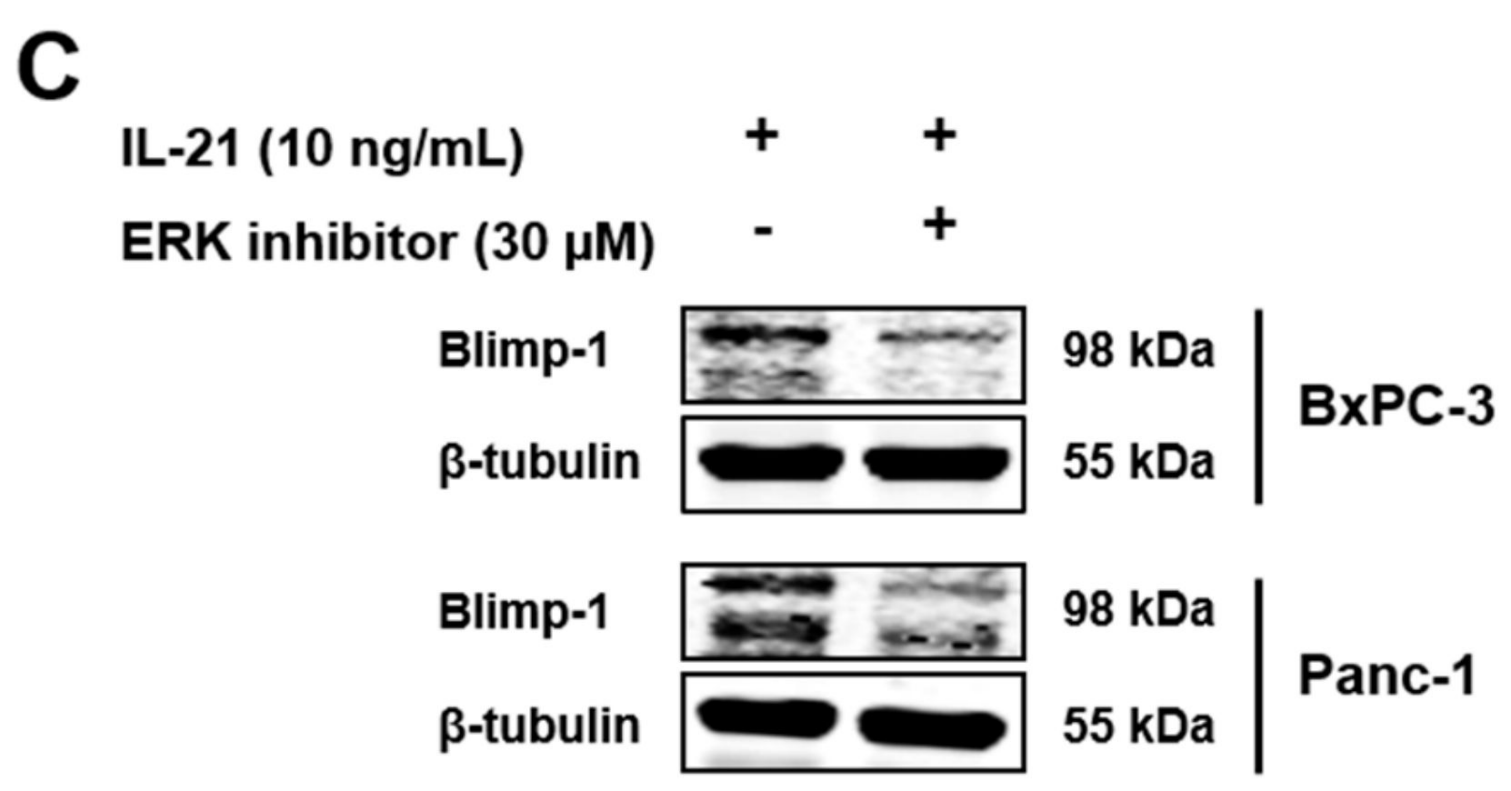
IL-21 upregulates Blimp-1 in an ERK-dependent manner. BxPC-3 and Panc-1 were incubated with IL-21 (10 and 25 ng/mL) for 24 h. (**A**) Treatment with IL-21 upregulates Blimp-1 expression. One of six independent experiments is shown. (**B**) Quantification of IL-21-dependent Blimp-1 upregulation (IL-21: 10 ng/mL). * *P* < 0.05. Wilcoxon rank-sum test. (**C**) ERK inhibitor prevents IL-21-dependent Blimp-1 upregulation. One of five independent experiments is shown.

Moreover, after a 24 h IL-21 stimulation, a significant induction of Blimp-1 protein expression was seen for BxPC-3 and Panc-1 (35% increase for BxPC-3 and 62% increase for Panc-1, average calculated from n = 6 independent experiments) (Figure 5A,B). Prolonged culture of IL-21 did not result in a further increase; in line with the finding that mRNA for *PRDM1* was already induced within 2 h (BxPC-3: 46.8%, Panc-1: 47.2%; n = 3) (Supplementary Figure S2A,B). Upregulation of Blimp-1 was reduced when cells were cultivated in the presence of an ERK inhibitor (Figure 5C).

### 3.3. IL-21 Upregulates Blimp-1 in an Avian Xenograft Model

To verify the functionality of the IL-21 receptor in vitro, we performed xenograft studies. The chicken egg is a naturally immunodeficient system that does not reject foreign tissue and supports the growth of xenograft tumors by host blood vessels and stroma [38]. Here, tumor cells were engrafted on the chorioallantoic membrane (CAM) of fertilized chicken eggs in the presence or absence of IL-21 at day 9 of chick development. At that time point, the blood vessel system of the embryo was dense enough to support tumor growth. Intravenous in ovo treatment was conducted twice, at day 12 and 16 (day 2 and 6 after cell engraftment). For both the vehicle control and IL-21 group, the same number of chick embryos survived until day 17 (13/50) when embryos were euthanized and the tumor was resected.

**Figure 6:**
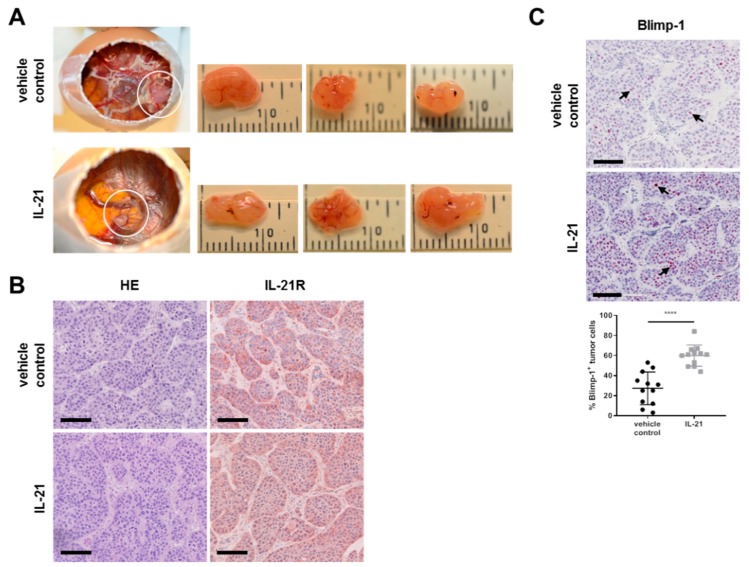
Treatment of avian xenografts with IL-21 upregulates Blimp-1 expression. (**A**) The three-dimensional growth of BxPC-3 cells transplanted on the CAM of fertilized chicken eggs and resected tumors (three replicates) from vehicle control and IL-21 were photographed at day 17 of embryonic development. Upper row for vehicle control; lower row for IL-21-treated (150 ng/mL) tumors. (**B**) HE and IL-21R (brown) IHC staining of vehicle control and IL-21-treated (150 ng/mL) resected xenograft tumors. (**C**) Comparison and quantification of Blimp-1 (nuclei: red) expression of vehicle control and IL-21-treated (150 ng/mL) xenograft tumors. **** *P* < 0.0001. Unpaired *t*-test.

As shown in Figure 6A,B, tumor cells were engrafted on the CAM and expressed the IL-21 receptor. Furthermore, in the IL-21-treated group, expression of Blimp-1 was significantly increased (>two-fold upregulation (27.33 vs 59.92%)) (Figure 6C).

### 3.4. IL-21 Enhances Invasion of Pancreatic Tumor Cells In Vitro

**Figure 7:**
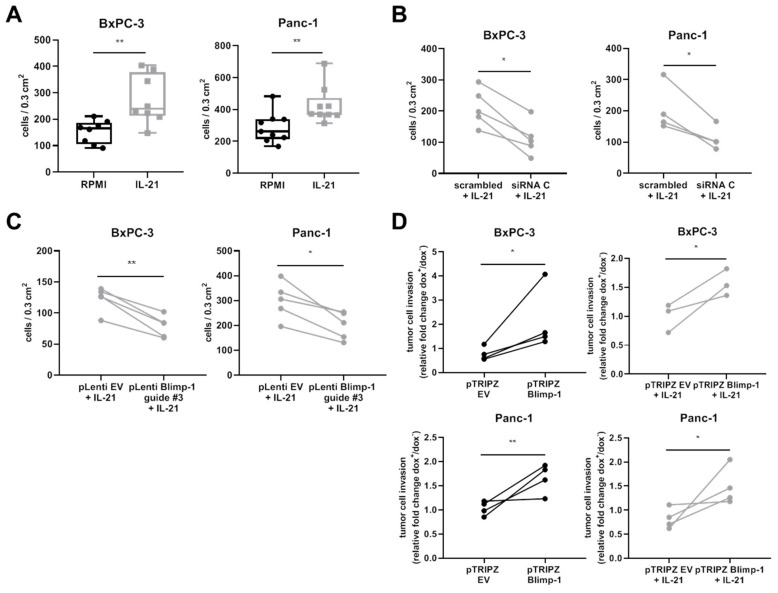
IL-21 promotes cell invasion in human PDAC cell lines in a Blimp-1-dependent manner. (**A**) Invasion of either untreated (RPMI) or IL-21-treated (10 ng/mL) BxPC-3 and Panc-1 cells was determined after 48 h. (**B**) Invasion of BxPC-3 and Panc-1 cells transfected with either a scrambled siRNA or a *PRDM1*-targeting siRNA (siRNA C) for Blimp-1 protein knockdown after 48 h of IL-21 (10 ng/mL) treatment. (**C**) Invasion of BxPC-3 and Panc-1 transfected with pLenti EV or pLenti Blimp-1 construct for CRISPR/Cas9-mediated Blimp-1 knockout after 48 h of IL-21 (10 ng/mL) treatment. (**D**) Invasion of BxPC-3 and Panc-1 transfected with a dox-inducible pTRIPZ EV or pTRIPZ Blimp-1 construct for Blimp-1 overexpression. Relative fold change of invaded cells treated with (dox^+^) and without dox (dox^−^) (1 µg/mL). Left panel: invasion of untreated (RPMI) cells, right panel: invasion of IL-21-treated (10 ng/mL) cells * *P* < 0.05 and ** *P* < 0.01. Unpaired *t*-test.

To answer the question whether stimulation of cells via IL-21R alters cell function, effects on invasion were tested. Here, in vitro invasion of cells into a 3-D Matrigel matrix was significantly enhanced in IL-21-treated cells (Figure 7A).

siRNA-mediated knockdown of Blimp-1 (Supplementary Figure S3) significantly decreased invasion of pancreatic cancer cells, as evaluated by an unpaired *t*-test (Figure 7B). Another approach was conducted using CRISPR/Cas9-mediated knockout (KO) of Blimp-1. Efficacy of guides was tested using a T7 endonuclease assay. Here, guide #3 was evaluated as a positive candidate showing editing in T7 assay (Supplementary Figure S4). A decrease in invasion could be confirmed with the CRISPR/Cas9 KO approach (Figure 7C). These experiments indicate that the IL-21 effect was at least partly dependent on Blimp-1.

In line with the data, dox-inducible Blimp-1 overexpression (Supplementary Figure S5) showed a significantly increased tumor cell invasion. IL-21 stimulation, however, had no additional effect on increased invasion under these experimental conditions (Figure 7D).

### 3.5. Effect of IL-21 on Proliferative Acivity and Growth of Tumor Cells

**Figure 8:**
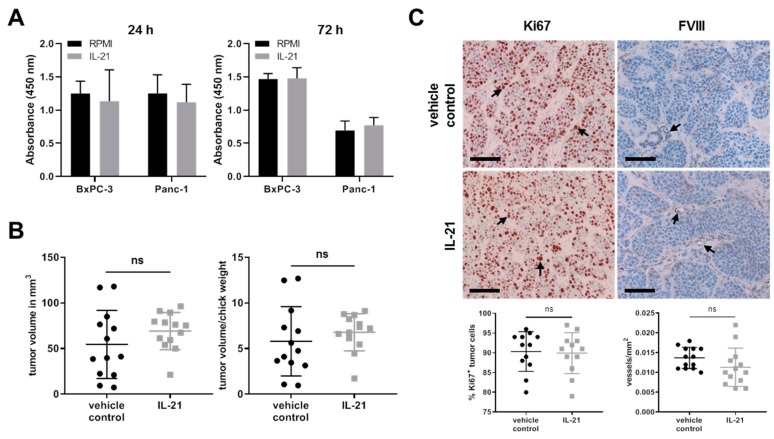
IL-21 treatment has no profound effect on tumor cell proliferation. (**A**) Proliferation of BxPC-3 and Panc-1 was measured by a BrdU incorporation method at the indicated time points. Data from four independent experiments are shown. (**B**) Quantification of tumor volume and the ratio of tumor volume to chick weight. Unpaired *t*-test. (**C**) Comparison and quantification of Ki67 (nuclei: brown) and FVIII (tubular structure: brown) expression of vehicle control and IL-21-treated (150 ng/mL) xenograft tumors. Arrows indicate positive cells. Black bar: 100 µm. Unpaired *t*-test.

Effects on proliferation upon stimulation of cells via IL-21R were analyzed using a BrdU assay. IL-21 (10 ng/mL) did not significantly promote proliferative activity within 72 h in vitro (Figure 8A). In the avian xenograft, the resected tumors differed with regard to size within the groups; there was, however, only a minor but not significant difference between the IL-21-treated and vehicle control group (Figure 8B). In line with these findings, there was no difference in proliferation (Ki67) or neovascularization (FVIII) in the avian xenograft, as analyzed by immunohistochemistry (Figure 8C).

## 4. Discussion

Tumors elicit an immune response with intend to eliminate tumor cells. T cells, however, may develop means to escape the immune attack, and rather—paradoxically—the infiltrating immune cells may contribute to tumor progression, for example by releasing cytokines. Cytokines and their respective receptors are essential for the communication among immune cells but many tumors, including those of non-hematopoietic origin, may acquire cytokine receptors and in turn use cytokines for their own benefit [40].

In this context, numerous cytokines are considered either ‘anti-neoplastic’ or, quite the opposite, as ‘tumor-promoting’. Before placing a cytokine in either category, a few caveats have to be considered: aberrant expression of functional cytokine receptors on non-immune cells, such as tumor cells; the heterogeneity of the inflammatory tumor microenvironment and the awareness that there is no significant intratumor inflammation in the widely used tumor xenograft models due to the immune incompetence of the host. Moreover, the majority of cytokines are pleiotropic. Some are potent drivers of chronic inflammatory processes and hence also of inflammatory carcinogenesis [30,41,42]. Also, the histological phenotype of given tumors (epithelial versus mesenchymal versus lymphatic) may affect the reactivity towards a particular cytokine. Therefore, in this study, we used a variety of methods, including tissue samples, in vitro cell assays using cells of different origin and differentiation status and an in vivo xenograft model to determine the role IL-21 in PDAC.

Previous data, by us and others, found that in PDAC infiltrates of particular T helper cell populations were associated with tumor progression and poor survival [9,10,43], leading to the question of underlying mechanisms. In patients with particularly aggressive disease, high numbers of Th cells, activated via an alternative p38 MAPK kinase pathway and producing high levels of TNF-α, IL-17A and IL-21, are present in tissue [10]. Because IL-21 is a major cytokine released by Th17 helper cells, the latter prevalent in PDAC, the infiltration of IL-21^+^ immune cells and the expression of the respective receptor in pancreatic tumor tissue was analyzed. Dense intratumor infiltrates of IL-21^+^ immune cells were associated with poor prognosis, in line with data linking dense infiltrates of Th17 with disease progression in PDAC [9,10]. Healthy pancreatic tissue does not express significant amounts of the corresponding receptor IL-21R. However, in the majority of tissues from PDAC patients, IL-21R was found on tumor cells, though to a varying degree, as were IL-21^+^ infiltrates. The observation that patients with dense IL-21^+^ infiltrates showed a shorter survival, prompted us to extend our studies to possible molecular mechanisms. In vitro data with pancreatic tumor cells confirmed expression of IL-21R on pancreatic tumor lines. The cells could be triggered by recombinant IL-21. Phosphorylation of key tumor signaling pathways ERK and STAT3 was seen, in line with data on IL-21 signaling pathways in other cells, e.g., lymphocytes [18,44,45,46,47]. Furthermore, IL-21 induced the transcription factor Blimp-1 in an ERK-dependent manner, again in line with previous data by others describing Blimp-1 as a down-stream target of the ERK pathway [48,49,50].

Blimp-1, initially detected as a major regulator of B cell survival [51,52], has now evolved as a potent and divergent regulator not only of B cells, but of other immune cells as well [53,54,55,56]. Of note, Blimp-1 expression is not restricted to hematopoietic cells but is also found in breast cancer cells [50], lung cancer cells [57], or inflammatory synoviocytes [58], where it is upregulated by various factors, derived from the micromilieu and associated with disease progression.

We found upregulation of Blimp-1 in response to IL-21 in cell lines and the in vivo xenograft model. The close association of Blimp-1 with the IL-21R in PDAC tissue suggests that also in its natural environment IL-21 induces Blimp-1. High Blimp-1 expression, and concurrent high expression of both, IL-21R and its downstream target Blimp-1, were associated with poor survival of PDAC patients, implicating IL-21 in tumor progression. As a possible underlying cellular process, we observed an enhanced capacity of IL-21 treated tumor cells to migrate into a 3-D gel matrix, a method imitating invasive cell growth. On a molecular level, the enhanced invasive capacity of the tumor cells could be attributed to Blimp-1, showing that Blimp-1 overexpression increased invasion, whereas its knockdown decreased IL-21-mediated tumor cell invasion. 

Taken together, the data indicate that in pancreatic tumor cells IL-21 induces capacities required for metastasis formation in a Blimp-1-dependent manner. Given the fact that IL-21-mediated Blimp-1 regulation is not restricted to tumor cells and is rather a mechanism found in immune cells [59], this is an example of how tumor cells can hijack immune-related pathways.

Blimp-1 tissue expression correlated with a higher probability of developing metastasis in a subtype of breast cancer patients [50]. These data are in line with observations in a genetically-engineered PDAC mouse model, where Blimp-1 was identified as a major ‘driver’ for metastasis [60]. Hypoxia within the tumor was found to induce Blimp-1 in this model. Effects of the inflammatory mediators in tumor microenvironment, however, were not investigated in this study. In advanced tumors, hypoxia and consequently upregulation of genes induced by hypoxia are to be expected. While we cannot rule out hypoxia as contributing factors in vivo, our in vitro data clearly indicate that IL-21 induces Blimp-1. Under our experimental conditions, we did not see effects of IL-21 on tumor growth or vascularization.

Aside from the activation of ERK and Blimp-1, a further—not necessarily directly associated— driver of tumor progression is STAT3. Enhanced STAT3 activity, as it is seen in response to various cytokines and as we have shown also in response to IL-21, is driving multiple pro-oncogenic functions in a wide variety of tumors (reviewed in [61,62]). For PDAC, in an experimental set up, a critical role for STAT3 was seen [63], which, however, appears to be operative in early events of tumor manifestation.

## 5. Conclusions

In summary, high numbers of intratumor IL-21^+^ infiltrates, aberrant expression of IL-21R, and its downstream target Blimp-1 in pancreatic tumor cells are associated with disease progression of PDAC. On a molecular level, invasiveness of tumor cells, a key factor required for metastasis, was affected by IL-21. Taken together, our data support the concept that the proinflammatory tumor microenvironment is crucially involved in tumor progression.

## Supplementary Materials

The following are available online at https://www.mdpi.com/2073-4409/8/9/1104/s1, Supplementary Figure S1: GATA3 expression in human PDAC and healthy pancreas tissue samples, Figure S2: Prolonged culture of IL-21, Figure S3: siRNA-mediated knockdown of Blimp-1, Figure S4: T7 endonuclease assays on Blimp-1 sites, Figure S5: Dox-inducible Blimp-1 overexpression, Supplementary Table S1: Oligonucleotides for sgRNA cloning, Table S2: Primers for cloning, Table S3: Primers for T7 endonuclease analysis.
