# Supplementary material for: Interleukin 21 Receptor/Ligand Interaction Is Linked to Disease Progression in Pancreatic Cancer
Source: Cells. 2019 Sep 18;8(9):1104. doi: 10.3390/cells8091104 (PMC6770770; doi:10.3390/cells8091104)
Supplement: Supplementary file 1 [file cells-08-01104-s001.pdf]

Supplementary Figures

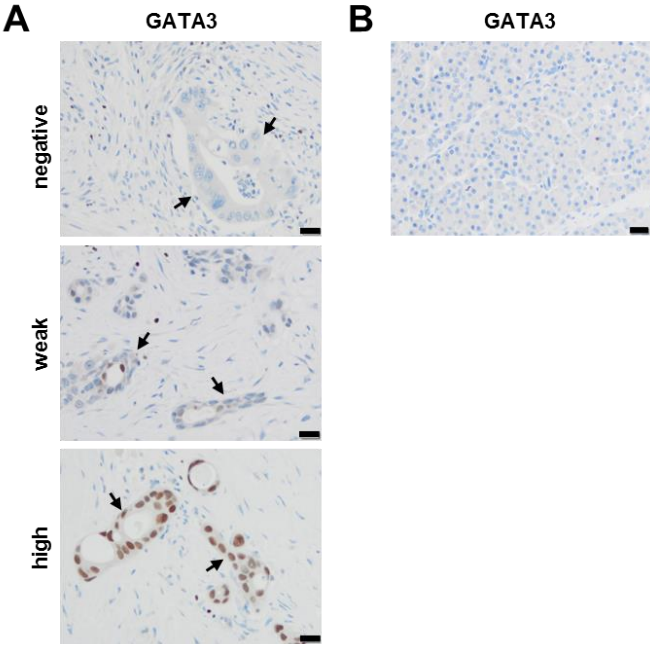

Supplementary Figure S1. GATA3 expression in human PDAC and healthy pancreas tissue samples. (**A**) Examples of negative, weak and high expression of GATA3 (brown) in human PDAC tissue. Black arrows: tumor cells. (**B)** Example of GATA3 expression in healthy pancreas tissue**.** Black bar: 20 µm.

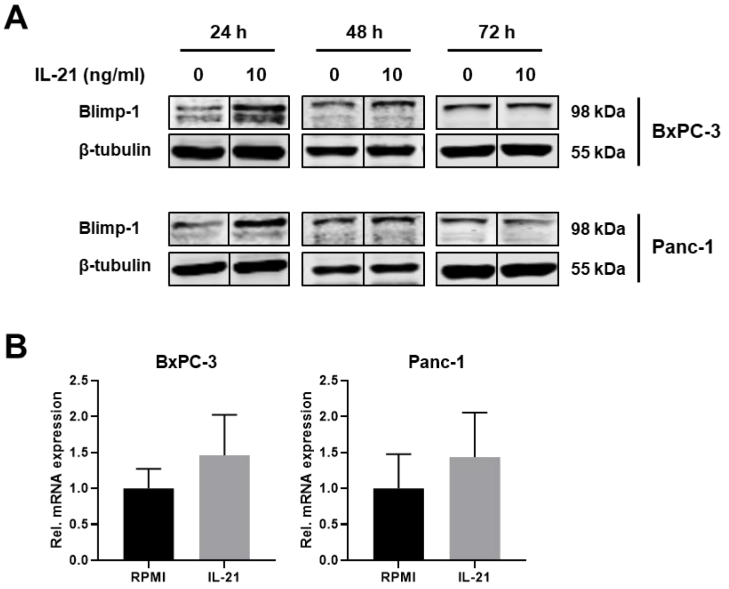

Supplementary Figure S2. Prolonged culture of IL-21 does not further increase Blimp-1 protein expression. (**A**)**:** Immunoblot showing Blimp-1 expression after 24 h, 48 h and 72 h of IL-21 treatment (10 ng/mL). (**B**): *PRDM1* mRNA expression in BxPC-3 and Panc-1 after 2 h of IL-21 treatment (10 ng/mL).

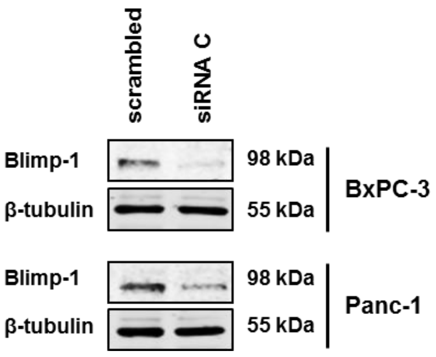

Supplementary Figure S3. siRNA-mediated knockdown of Blimp-1. Immunoblot showing Blimp-1 knockdown in BxPC-3 and Panc-1 cells after transfection with either a scrambled control or a *PRDM1*-targeting siRNA (siRNA C).

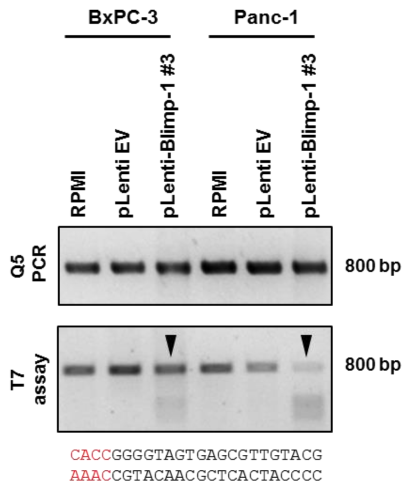

Supplementary Figure S4. T7 endonuclease assays on Blimp-1 sites. sgRNA *PRDM1* guide #3 shows editing in T7 endonuclease assay as indicated by black arrow heads.

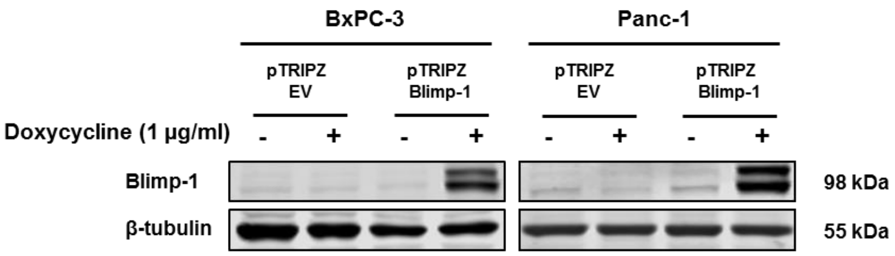

Supplementary Figure S5. Dox-inducible Blimp-1 overexpression. Immunoblot showing dox-inducible Blimp-1 overexpression (1 µg/mL, 48 h of treatment) of a pTRIPZ EV and a pTRIPZ Blimp-1 construct.

Supplementary Tables

Supplementary Table S1. Oligonucleotides for sgRNA cloning.

sgRNA Oligo	Sequence
PRDM1 guide #3 top	CACCGGGGTAGTGAGCGTTGTACG
PRDM1 guide #3 bot	AAACCGTACAACGCTCACTACCCC

Supplementary Table S2. Primers for cloning.

Primer	Sequence
pTRIPZ_AgeI_Blimp1 For	AGAGCTCGTTTAGTGAACCGTCAGATCGCAGCCACCCATCACCATCACCATCACGGG
pTRIPZ_MluI_Blimp1 Rev	GGCGCCAAAACCCGGCGCGGAGGCCACGCGTCAAGGGTCCATTGGTTCAAC

Supplementary Table S3. Primers for T7 endonuclease analysis.

Primer	Sequence
PRDM1 NCBI3.2 for	CCCCTCTGCCAGAAGACTTT
PRDM1 NCBI3.2 rev	CATCGCTGCTGAGGTAGCTT
